# Iron chelators as a therapeutic option for Alzheimer’s disease—A mini-review

**DOI:** 10.3389/fragi.2023.1234958

**Published:** 2023-08-02

**Authors:** Oliver Daniel Schreiner, Thomas Gabriel Schreiner

**Affiliations:** ^1^ Grigore T. Popa University of Medicine and Pharmacy, Iasi, Romania; ^2^ Medical Oncology Department, Regional Institute of Oncology, Iasi, Romania; ^3^ Faculty of Electrical Engineering and Information Technology, Gheorghe Asachi Technical University of Iasi, Iasi, Romania; ^4^ Faculty of Medicine, University of Medicine and Pharmacy “Carol Davila”, Bucharest, Romania

**Keywords:** aging, metabolism and redox biology chelation, Alzheimer’s disease, neurodegenearation, iron homeostasis, ferroptosis

## Abstract

Neurodegenerative disorders, particularly Alzheimer’s disease (AD), remain a great challenge regarding the finding of effective treatment, one main reason being the incomplete understanding of their etiology. With many intensely debated hypotheses, a newer approach based on the impact of iron imbalance in sustaining neurodegeneration in the central nervous system becomes increasingly popular. Altered iron homeostasis leads to increased iron accumulation in specific brain areas, explaining the clinical picture of AD patients. Moreover, growing evidence sustains the significant impact of iron metabolism in relationship to other pathological processes encountered in the AD-affected brain, such as the amyloidogenic pathway, chronic inflammation, or oxidative stress. In this context, this mini-review aims to summarize the novel data from the continuously expanding literature on this topic in a didactic manner. Thus, in the first part, the authors briefly highlight the most relevant aspects related to iron absorption, transport, regulation, and elimination at the cerebral level, focusing on the role of the blood-brain barrier and the newer concept of ferroptosis. Subsequently, currently available iron chelation therapies are discussed, including an overview of the most relevant clinical trials on this topic. In the final part, based on the latest results from *in vitro* and *in vivo* studies, new research directions are suggested to enhance the development of effective antidementia therapies.

## 1 Introduction

Neurodegenerative disorders (NDDs) include a heterogenous group of pathologies, with Alzheimer’s disease (AD) being the most frequent, according to present epidemiological data (Cui et al., 2020; Gustavsson et al., 2023). NDDs have some common features that differentiate them from other non-communicable diseases in humans, despite having heterogeneous clinical manifestations, such as predominant motor symptomatology in Parkinson’s disease (Moustafa et al., 2016) or, more important cognitive and behavioral deficits in frontotemporal dementia (Johnen and Bertoux, 2019). NDDs, particularly AD, are a challenge for the clinician because of several reasons: the increasing prevalence in the aging population (Logroscino et al., 2022), the incomplete understanding of the underlining pathophysiology (Wilson et al., 2023), and the absence of an effective or curative treatment despite the immense number of clinical trials conducted in recent years (Mortberg et al., 2022). Because of the lack of effective treatment, one promising first step for ensuring antidementia therapeutic advancements remains the return to basic pathophysiological processes that could explain the onset and evolution of AD (Vaz and Silvestre, 2020; Mehkri et al., 2022). Currently, many debated hypotheses incompletely describe the occurrence of AD; the most acknowledged ones focus on the pathological accumulation of misfolded proteins such as amyloid beta (Aβ) (Karran and De Strooper, 2022), the negative influence of Tau protein hyperphosphorylation (Rawat et al., 2022), the role of ApoE4 and its related protein (Raulin et al., 2022), the negative impact of reactive oxygen species (Bhatt et al., 2021), chronic inflammation involving neurons and glial cells in the central nervous system (CNS) (Xie et al., 2022), and the role of the blood-brain barrier (BBB) disruption in the evolution of the disease (Hussain et al., 2021). A recent approach is based on the role of iron in the healthy brain and the consequences of dysregulated iron metabolism concerning the cellular and molecular dysfunction encountered in AD (Peng et al., 2021). Although the research on this topic is still in its infancy, with many unknowns and incompletely explained mechanisms, this concept could become a linking point for the other accepted theories on neurodegeneration (Spotorno et al., 2020; Ward et al., 2022). Moreover, the “ferroptosis hypothesis of AD” is a valuable source for developing new drugs that might be at least effective adjuvant treatment, if not promising principal therapies (Nikseresht et al., 2019). With growing data on iron pathophysiology in the AD-affected brain and the emergence of novel iron chelation-based therapies, there is a great need to summarize the current knowledge of expanding literature in the field. In this context, this mini-review aims to offer a succinct and practically-oriented overview of the presently available iron chelators used in the clinical areas, with potential interest for AD treatment, and a summary of the theoretical data on iron metabolism. Discussed topics include iron absorption, transport, regulation, and elimination at the CNS level; the relationship between iron metabolism and other pathological processes encountered in AD, such as protein misfolding, inflammation, oxidative stress, and the alteration of the blood-brain barrier; the newer concept of ferroptosis is also covered.

## 2 Systemic and cerebral iron metabolism in physiological conditions in humans

Iron, an essential trace metal in the human body, plays important roles in many physiological processes, such as redox reactions (Koppenol and Hider, 2019), metabolic pathways modulation (Phelan et al., 2018), DNA synthesis and repair (Carter et al., 2022), and mitochondrial energy generation (Onukwufor et al., 2022). These biological processes are possible because of iron’s unique chemical characteristics, especially its ease of maintaining a dynamic balance between the bivalent and the trivalent forms (Yiannikourides and Latunde-Dada, 2019). Further, a clear differentiation between iron homeostasis in the CNS *versus* the periphery was made to highlight the importance of the BBB as the main boundary between the two distinct compartments. In physiological conditions, there are 3–5 g of iron in the human body (Abbaspour et al., 2014); this constant quantity is provided by two sources, absorption via intestinal cells from food (Fuqua et al., 2012) and release from macrophages (Sukhbaatar and Weichhart, 2018). The complex molecular mechanisms involved in enteral iron absorption are reviewed elsewhere (Gulec et al., 2014). A good understanding of the roles played by the duodenal cytochrome-b-like protein (DCYTB) (Lane et al., 2015) located at the apical side of the intestinal cell or by ferroportin (Nemeth and Ganz, 2021), located at the basal side of the enteric cell, is mandatory to develop therapeutic means which could enhance or limit iron absorption according to individual needs. Once in plasma, iron can bind to transferrin (Gkouvatsos et al., 2012), subsequently fixes to specific membrane receptors, and enters intracellularly via clathrin-mediated endocytosis (Gammella et al., 2021). Two main iron storage compartments exist in the periphery, hepatocytes and macrophages, storing iron mainly in the form of ferritin (Recalcati and Cairo, 2021). Additionally, iron can also be found in the formation of myoglobin (Elkholi et al., 2022), cytochrome (Misslinger et al., 2017), and other iron-regulated enzymes (Poulos, 2014).

An interesting aspect is related to the regulatory mechanisms, with iron regulatory proteins (IRP) as the main players in maintaining intracellular iron homeostasis (Zhang et al., 2014). IRP1 and IRP2 act like iron sensors (Zhang et al., 2014). Still, their activity is also modulated by other molecules, such as oxygen and nitrous oxide (Luo et al., 2011), explaining the tight correlation between iron metabolism and oxidative stress (Cairo and Recalcati, 2007). On the other hand, the liver is a fine regulator of the systemic concentration of iron via the secretion of hepcidin (Vela, 2018), while macrophages are capable of increasing the iron level in the systemic circulation through the phagocytosing of aging red blood cells (Recalcati and Cairo, 2021). Finally, recent studies showed the impact of gut microbiota in modulating iron absorption (Malesza et al., 2022). Besides the direct, local mechanisms of the microbiome, a key role is thought to be also played by the gut-brain axis, with indirect evidence resulting from clinical trials conducted in psychiatric patients (Fernández Real et al., 2019). Knowing in detail the mechanisms that ensure iron homeostasis in humans is of interest from a clinical point of view: genetic diseases remain an important chapter in pathology, with numerous genetic mutations currently known to lead to iron overload syndromes (such as hereditary hemochromatosis) with significant systemic impact (Piperno et al., 2020).

When studying iron absorption at the CNS level, mechanisms are similar to the gastrointestinal endothelial tissue. However, the endothelial layer forming the wall of the cerebral blood vessels has some particularities. The BBB, a unique and highly selective structure in the human brain (Alahmari, 2021), is responsible for ensuring the protection of the sensitive cerebral tissue against external toxic factors (Pandit et al., 2020), meaning that free circulating and transferrin-bound iron must pass via the brain microvascular endothelial cell (BMEC) by specific mechanisms. Several explanations can be found in the literature, The roles of ferroportin 1 (Mezzanotte et al., 2022), ceruloplasmin (Ryan et al., 2019), hephaestin (Zacchi et al., 2021), and holo-transferrin (Baringer et al., 2022) have been proposed to explain iron-transferrin complex internalization, while free, unbound iron is suspected to enter the neuron via transcytosis, after binding to heavy-chain ferritin (H-ferritin) (Mesquita et al., 2020) or lactoferrin (Kell et al., 2020).

The intraneuronal metabolism of iron is not significantly different from the metabolism of other cells found in the periphery; however, there should be noted an overexpression of transferrin 1 receptors on the surface of the neurons (Menon et al., 2019) and an increased age-dependent cerebral level of ferritin (Ficiarà et al., 2022). Moreover, besides neurons, glial cells and microglia are also involved in regulating iron cerebral levels (Xu et al., 2018), ensuring neural protection against iron toxicity (Nnah and Wessling-Resnick, 2018). Iron is involved in the myelination of oligodendrocytes, the most iron-rich cells in the brain (Khattar et al., 2021), this finding being additional proof of the multitude of physiological roles iron exerts at the CNS level.

## 3 Iron dyshomeostasis in Alzheimer’s disease

Although the pathological accumulation of misfolded proteins is thought to be the central feature of AD pathogenesis, alterations in iron homeostasis with subsequent iron accumulation should also be considered as the driver of AD pathology (Liu et al., 2018). Proof for this new research direction is the multitude of studies that have demonstrated abnormally high levels of iron in key brain regions, such as the hippocampus or the frontal cortex, beginning with the preclinical stages of AD (Tran et al., 2022; Lin et al., 2023). Additionally, the cerebral iron load was correlated with the severity of the symptoms (especially cognitive deficit) (Spence et al., 2020); it was also considered a reliable predictor of the evolution of the disease (Ayton et al., 2020a).

Several hypotheses try to explain iron dyshomeostasis in AD. Firstly, the close interaction with amyloid plaques and neurofibrillary tangles should be noted (Liu et al., 2018; Wang et al., 2022). On the one hand, the anatomopathological (Tran et al., 2022) and imaging studies (Gong L. et al., 2019) showed an increased concentration of iron in the areas rich in Aβ accumulations and also in cortical areas rich in Tau protein accumulations. On the other hand, the disruption of the normal iron metabolism at the cerebral level, with the alteration of IRP, supports the amyloid cascade by upregulating amyloid precursor protein (APP) and by modulating alpha and beta-secretases, two key enzymes involved in APP degradation (Gong N. J. et al., 2019). However, there is no proof up to the present of a direct impact of iron accumulation on senile plaque formation. It seems that iron accumulation occurs concomitantly with amyloid accumulation in the brain as two synergic processes that lead to neurodegeneration (Peters et al., 2015). It remains to be determined if iron imbalance precedes and could trigger senile plaque formation. Regarding the effect of iron dyshomeostasis on Tau hyperphosphorylation, a direct correlation was observed between increased iron intake, cognitive deficit, and abnormal accumulation of Tau protein, with insulin signaling as the main molecular mechanism (Wan et al., 2019).

However, the impact of iron at the brain level is much more closely related to oxidative stress, as iron is an essential factor in numerous redox reactions resulting in the generation of reactive oxygen species (ROS) (Zhang et al., 2022). At the same time, excess iron reduces antioxidant mechanisms, ultimately leading to ferroptosis and neuronal loss (Mancardi et al., 2021). Ferroptosis, a type of programmed cell death first described in 2012 (Dixon et al., 2012), involves lipid peroxidation generated by the iron overload, leading to cell swelling, mitochondrial dysfunction, nuclear chromatin condensation, and, finally, cellular membrane rupture (Han et al., 2020). Various studies have shown a correlation between the accumulation of iron in the brain which means increased ferroptosis and aging (Coradduzza et al., 2023), cerebrovascular diseases (Liu et al., 2022), and neurodegenerative diseases (including AD) (Ma et al., 2022). However, the exact molecular mechanisms of iron-induced neurodegeneration remain incompletely known. Recent research focused mainly on the roles of glutathione peroxidase 4 (GPX4) (Cardoso et al., 2017) and glutamate/cystine antiporter (xCT) (Lane and Lin, 2023) in the pathogenesis of AD in both animal models and humans, an overview of the currently accepted mechanism being schematized in the work of Wang et al., 2022.

Finally, iron can also lead to neurodegeneration by regulating glial cells, more precisely by activating microglia (Long et al., 2022) and astrocytes (Codazzi et al., 2015). Microglia are strongly reactive to iron exposure, and, when activated, they produce pro-inflammatory cytokines, thus facilitating the accumulation of Aβ (Cai et al., 2022). Moreover, activated microglia support the additional accumulation of iron in the brain through a positive feedback mechanism (Kenkhuis et al., 2021). On the other hand, in the first phases of inflammation, astrocytes are less reactive compared to neurons or microglia; however, in the later stages, astrocytes also generate inflammatory mediators, subsequently supporting oxidative stress, the chronic inflammatory status, and the pathological accumulation of misfolded proteins such as Tau and Aβ (Monterey et al., 2021). Astrocyte activation by pathological iron accumulation is an indirect link between iron and the BBB, as chronic neuroinflammation leads to BBB disruption, which means increased permeability and sustained neuronal damage. Yet, the astrocyte-heavy metal link is bidirectional, with astrocytes demonstrated to control iron and other metal ions concentration in the brain parenchyma via specific transporters (Li et al., 2021). This also explains why astrocyte dysfunction is strongly related to cerebral iron imbalance in neurodegenerative disorders, including AD.

## 4 Iron chelators in clinical practice

With increasing evidence related to the role of iron in the pathogenesis of AD, iron chelation could be a potentially effective therapeutic approach. This therapy is already successfully used in iron overload syndromes such as sickle cell disease, major beta-thalassemia, and rare disorders of iron-transporting proteins (Bruzzese et al., 2023). In this context, there are drugs approved for daily clinical use, their relevant characteristics being summarized in Table 1.

**TABLE 1:** Currently used iron chelators in clinical practice.

Iron chelator	Administration protocol	Advantages	Disadvantages	Crossing the blood-brain barrier	Special regulations by country/region
Deferasirox (DFX)	Unique dose of 20–30 mg/kg/day, oral administration	Once daily administration	Important side effects: potentially fatal gastrointestinal hemorrhage, renal and hepatic toxicity	Via nanocarrier	FDA and EMA approved.Adults with chronic iron overload (in Europe also in children 6 years and older)
			High costs		
Deferiprone (DFP)	75 mg/kg/day (divided into 2–3 doses/day), oral administration	Most effective in cardiac iron excretion	Important side effects: gastrointestinal symptoms, hepatic toxicity, neutropenia, and agranulocytosis	Yes	Available in the United States, Canada, and other countries Second-line therapy in adult patients with thalassemia major
			Frequent (weekly) blood count monitoring		
			Important side effects		
Deferoxamine (DFO)	25–50 mg/kg/day, subcutaneous or intravenous infusion	More than 30 years of experience	visual and auditory neurotoxicity, gastrointestinal symptoms, increased	Yes	FDA and EMA approved.First-line therapy for hemochromatosis
	over 8–12 h		risk of infections		
	5 days/week		(mucormycosis) Decreased compliance because of the administration route		

Deferoxamine (DFO) is the most well-known iron chelator, with more than 3 decades of clinical experience (Parker et al., 2023). However, the visual and ototoxicity (Derin et al., 2017) and potentially decreased compliance due to the subcutaneous/intravenous administration protocol (Yarali et al., 2006) opened the pathway for developing newer drugs. Deferasirox (DFX) and deferiprone (DFP) are two orally administered iron chelators that are more patient-friendly regarding the potential side effects (Entezari et al., 2022). However, higher costs for DFX and weekly blood monitoring in the case of DFP can limit their use (Ravalli et al., 2022). Finally, there is also the possibility to administer the abovementioned drugs in combination, however, despite being safe for patients, the exact benefits of this approach are incompletely studied (Origa et al., 2022).

## 5 Experimental research and future trends

Besides the already approved drugs, increasing research is conducted on novel therapeutic alternatives. Hydroxybenzyl ethylenediamine (HBED) has been longly proposed as a potential iron chelator (Samuni et al., 2001), but apart from a few incipient studies, the lack of sufficient data prevented its approval for routine clinical use. Ascorbic acid (vitamin C) is another relevant compound intensely studied in the last years, both because ascorbic acid is directly involved in redox reaction modulation and because the interaction between vitamin C and iron modulates many metabolic pathways (Kontoghiorghes et al., 2020). The main limitation is related to the weak capacity of vitamin C in chelating iron, having a low efficacy in iron elimination, and explaining why vitamin C is mainly recommended as adjunctive therapy (Elalfy et al., 2016).

Some compounds/drugs, initially designed to act on different molecular pathways, demonstrated also an effective iron-chelation feature. One good example is clioquinol, an antifungal and antiprotozoal drug, that demonstrated iron chelation characteristics in several conditions, such as pulmonary fibrosis (Zhu et al., 2021), Parkinson’s disease and AD (Nuñez and Chana-Cuevas, 2018). Similarly, antioxidant medications such as vitamin E, alpha-lipoic acid, and selenium are thought to interact indirectly with iron metabolism and could be potentially effective in treating AD.

Another group of potential adjuvant agents for iron depletion is represented by calcium channel blockers. The explanatory pathophysiologic mechanism is based on the blocking of the penetration of iron through calcium channels at the level of the heart, pancreas, and other organs, consequently possible prevention of visceral iron accumulation (Sun et al., 2020). A systematic review (Sadaf et al., 2018) highlighted the main reasons calcium channel blockers are still under scrutiny and not approved for clinical use: lack of sufficient clinical trials, no significant iron level reduction in organs and blood, and insufficient proof of safety. Thus, there is a high demand for new, larger randomized clinical trials to confirm the benefit of the abovementioned proposed iron chelating therapies.

Finally, apart from iron, other trace elements, such as copper, calcium, and zinc, seem to play relevant roles in CNS homeostasis and the pathophysiology of AD (Wang and Wang, 2017). Despite contradictory studies and many incompletely elucidated mechanisms, copper could become a valuable target for anti-dementia therapies (Ejaz et al., 2020). Moreover, future research should assess if any relevant link between iron and other trace elements exists and its impact on neurodegeneration.

## 6 Discussion

NDDs, particularly AD, represent an important burden worldwide, and with prevalence expected to sustainably grow in the next decades, the development of effective therapies is essential. The incomplete understanding of its etiology remains one of the main reasons for the slow therapeutic advancements for AD. The emergence during the last years of new hypotheses, complementary to the already established knowledge, should be noted, with the “ferroptosis hypothesis of AD” one good example.

Besides the important theoretical input, new theories related to AD pathogenesis are also valuable sources for new therapeutic approaches which might prove effective.

In this regard, iron chelators could be successfully used in AD treatment, as there are already three compounds approved for clinical use in patients with iron overload syndromes. Moreover, the possibility of using adjuvant therapies such as vitamin C or calcium channel blockers, and antioxidants such as vitamin E or alpha-lipoic acid, if proven efficient and safe, opens new perspectives for AD patients. At present, iron chelation in AD management remains an open topic, with only a few trials available. Worth to be mentioned is an old trial dating back to 1991, when intramuscularly chronically administered DFO (24 months, 5 days/week), showed a decrease in AD progression compared to the control group (Crapper McLachlan et al., 1991). Up to the present, no other randomized clinical trial on humans has delivered significant results, but we mention one notable, still-in-progress trial, that analyzes the effects of DFP in AD patients (Ayton et al., 2020b). Regarding DFX, only trials on animal models can be found when searching the literature (Kwan et al., 2022). With increasing theoretical background sustaining the impact of iron in AD pathogenesis, iron chelation should be more intensely studied as main or adjuvant therapy, in patients in different AD stages.

## References

[B1] Abbaspour N. Hurrell R. Kelishadi R. (2014). Review on iron and its importance for human health. J. Res. Med. Sci. 19 (2), 164–174. 24778671 PMC3999603

[B2] Alahmari A. (2021). Blood-brain barrier overview: Structural and functional correlation. Neural Plast. 2021, 6564585. 10.1155/2021/6564585 34912450 PMC8668349

[B3] Ayton S. Wang Y. Diouf I. Schneider J. A. Brockman J. Morris M. C. (2020a). Brain iron is associated with accelerated cognitive decline in people with Alzheimer pathology. Mol. Psychiatry 25 (11), 2932–2941. 10.1038/s41380-019-0375-7 30778133 PMC6698435

[B4] Ayton S. Woodward M. Ellis K. A. Lim Y. Y. Maruff P. T. Desmond P. M. (2020b). Deferiprone to delay dementia (the 3D trial). Drug Dev. 16, 59. 10.1002/alz.044107

[B5] Baringer S. L. Neely E. B. Palsa K. Simpson I. A. Connor J. R. (2022). Regulation of brain iron uptake by apo- and holo-transferrin is dependent on sex and delivery protein. Fluids Barriers CNS 19 (1), 49. 10.1186/s12987-022-00345-9 35689283 PMC9188189

[B6] Bhatt S. Puli L. Patil C. R. (2021). Role of reactive oxygen species in the progression of Alzheimer's disease. Drug Discov. Today 26 (3), 794–803. 10.1016/j.drudis.2020.12.004 33306995

[B7] Bruzzese A. Martino E. A. Mendicino F. Lucia E. Olivito V. Bova C. (2023). Iron chelation therapy. Eur. J. Haematol. 110 (5), 490–497. 10.1111/ejh.13935 36708354

[B8] Cai Y. Liu J. Wang B. Sun M. Yang H. (2022). Microglia in the neuroinflammatory pathogenesis of alzheimer's disease and related therapeutic targets. Front. Immunol. 13, 856376. 10.3389/fimmu.2022.856376 35558075 PMC9086828

[B9] Cairo G. Recalcati S. (2007). Iron-regulatory proteins: Molecular biology and pathophysiological implications. Expert Rev. Mol. Med. 9 (33), 1–13. 10.1017/S1462399407000531 PMC2811384 18053288

[B10] Cardoso B. R. Hare D. J. Bush A. I. Roberts B. R. (2017). Glutathione peroxidase 4: A new player in neurodegeneration? Mol. psychiatry 22 (3), 328–335. 10.1038/mp.2016.196 27777421

[B11] Carter A. Racey S. Veuger S. (2022). The role of iron in DNA and genomic instability in cancer, a target for iron chelators that can induce ROS. Appl. Sci. 12, 10161. 10.3390/app121910161

[B12] Codazzi F. Pelizzoni I. Zacchetti D. Grohovaz F. (2015). Iron entry in neurons and astrocytes: A link with synaptic activity. Front. Mol. Neurosci. 8, 18. 10.3389/fnmol.2015.00018 26089776 PMC4452822

[B13] Coradduzza D. Congiargiu A. Chen Z. Zinellu A. Carru C. Medici S. (2023). Ferroptosis and senescence: A systematic review. Int. J. Mol. Sci. 24 (4), 3658. 10.3390/ijms24043658 36835065 PMC9963234

[B14] Crapper McLachlan D. R. Dalton A. J. Kruck T. P. Bell M. Y. Smith W. L. Kalow W. (1991). Intramuscular desferrioxamine in patients with Alzheimer's disease. Lancet 337 (8753), 1304–1308. 10.1016/0140-6736(91)92978-b 1674295

[B15] Cui L. Hou N. N. Wu H. M. Zuo X. Lian Y. Z. Zhang C. N. (2020). Prevalence of alzheimer's disease and Parkinson's disease in China: An updated systematical analysis. Front. Aging Neurosci. 12, 603854. 10.3389/fnagi.2020.603854 33424580 PMC7793643

[B16] Derin S. Azık F. M. Topal Y. Topal H. Karakuş V. Çetinkaya P. U. (2017). The incidence of ototoxicity in patients using iron chelators. J. Int. Adv. Otol. 13 (1), 136–139. 10.5152/iao.2016.1852 27879229

[B17] Dixon S. J. Lemberg K. M. Lamprecht M. R. Skouta R. Zaitsev E. M. Gleason C. E. (2012). Ferroptosis: An iron-dependent form of nonapoptotic cell death. Cell 149 (5), 1060–1072. 10.1016/j.cell.2012.03.042 22632970 PMC3367386

[B18] Ejaz H. W. Wang W. Lang M. (2020). Copper toxicity links to pathogenesis of alzheimer's disease and therapeutics approaches. Int. J. Mol. Sci. 21 (20), 7660. 10.3390/ijms21207660 33081348 PMC7589751

[B19] Elalfy M. S. Saber M. M. Adly A. A. Ismail E. A. Tarif M. Ibrahim F. (2016). Role of vitamin C as an adjuvant therapy to different iron chelators in young β-thalassemia major patients: Efficacy and safety in relation to tissue iron overload. Eur. J. Haematol. 96 (3), 318–326. 10.1111/ejh.12594 26018112

[B20] Elkholi I. E. Elsherbiny M. E. Emara M. (2022). Myoglobin: From physiological roles to potential implications in cancer. Biochim. Biophys. Acta Rev. Cancer 1877 (3), 188706. 10.1016/j.bbcan.2022.188706 35247507

[B21] Entezari S. Haghi S. M. Norouzkhani N. Sahebnazar B. Vosoughian F. Akbarzadeh D. (2022). Iron chelators in treatment of iron overload. J. Toxicol. 2022, 4911205. 10.1155/2022/4911205 35571382 PMC9098311

[B22] Fernández Real J. M. Moreno-Navarrete J. M. Manco M. (2019). Iron influences on the Gut-Brain axis and development of type 2 diabetes. Crit. Rev. Food Sci. Nutr. 59 (3), 443–449. 10.1080/10408398.2017.1376616 28886251

[B23] Ficiarà E. Stura I. Guiot C. (2022). Iron deposition in brain: Does aging matter? Int. J. Mol. Sci. 23 (17), 10018. 10.3390/ijms231710018 36077413 PMC9456423

[B24] Fuqua B. K. Vulpe C. D. Anderson G. J. (2012). Intestinal iron absorption. J. Trace Elem. Med. Biol. 26 (2-3), 115–119. 10.1016/j.jtemb.2012.03.015 22575541

[B25] Gammella E. Lomoriello I. S. Conte A. Freddi S. Alberghini A. Poli M. (2021). Unconventional endocytosis and trafficking of transferrin receptor induced by iron. Mol. Biol. Cell 32 (2), 98–108. 10.1091/mbc.E20-02-0129 33236955 PMC8120689

[B26] Gkouvatsos K. Papanikolaou G. Pantopoulos K. (2012). Regulation of iron transport and the role of transferrin. Biochim. Biophys. Acta 1820 (3), 188–202. 10.1016/j.bbagen.2011.10.013 22085723

[B27] Gong L. Tian X. Zhou J. Dong Q. Tan Y. Lu Y. (2019a). Iron dyshomeostasis induces binding of APP to BACE1 for amyloid pathology, and impairs APP/Fpn1 complex in microglia: Implication in pathogenesis of cerebral microbleeds. Cell Transplant. 28 (8), 1009–1017. 10.1177/0963689719831707 30776900 PMC6728710

[B28] Gong N. J. Dibb R. Bulk M. van der Weerd L. Liu C. (2019b). Imaging beta amyloid aggregation and iron accumulation in Alzheimer's disease using quantitative susceptibility mapping MRI. NeuroImage 191, 176–185. 10.1016/j.neuroimage.2019.02.019 30739060

[B29] Gulec S. Anderson G. J. Collins J. F. (2014). Mechanistic and regulatory aspects of intestinal iron absorption. Am. J. Physiol. Gastrointest. Liver Physiol. 307 (4), G397–G409. 10.1152/ajpgi.00348.2013 24994858 PMC4137115

[B30] Gustavsson A. Norton N. Fast T. Frölich L. Georges J. Holzapfel D. (2023). Global estimates on the number of persons across the Alzheimer's disease continuum. Alzheimers Dement. 19 (2), 658–670. 10.1002/alz.12694 35652476

[B31] Han C. Liu Y. Dai R. Ismail N. Su W. Li B. (2020). Ferroptosis and its potential role in human diseases. Front. Pharmacol. 11, 239. 10.3389/fphar.2020.00239 32256352 PMC7090218

[B32] Hussain B. Fang C. Chang J. (2021). Blood-brain barrier breakdown: An emerging biomarker of cognitive impairment in normal aging and dementia. Front. Neurosci. 15, 688090. 10.3389/fnins.2021.688090 34489623 PMC8418300

[B33] Johnen A. Bertoux M. (2019). Psychological and cognitive markers of behavioral variant frontotemporal dementia-A clinical neuropsychologist's view on diagnostic criteria and beyond. Front. Neurol. 10, 594. 10.3389/fneur.2019.00594 31231305 PMC6568027

[B34] Karran E. De Strooper B. (2022). The amyloid hypothesis in alzheimer disease: New insights from new therapeutics. Nat. Rev. Drug Discov. 21 (4), 306–318. 10.1038/s41573-022-00391-w 35177833

[B35] Kell D. B. Heyden E. L. Pretorius E. (2020). The biology of lactoferrin, an iron-binding protein that can help defend against viruses and bacteria. Front. Immunol. 11, 1221. 10.3389/fimmu.2020.01221 32574271 PMC7271924

[B36] Kenkhuis B. Somarakis A. de Haan L. Dzyubachyk O. Ijsselsteijn M. E. de Miranda N. F. C. C. (2021). Iron loading is a prominent feature of activated microglia in Alzheimer's disease patients. Acta Neuropathol. Commun. 9 (1), 27. 10.1186/s40478-021-01126-5 33597025 PMC7887813

[B37] Khattar N. Triebswetter C. Kiely M. Ferrucci L. Resnick S. M. Spencer R. G. (2021). Investigation of the association between cerebral iron content and myelin content in normative aging using quantitative magnetic resonance neuroimaging. NeuroImage 239, 118267. 10.1016/j.neuroimage.2021.118267 34139358 PMC8370037

[B38] Kontoghiorghes G. J. Kolnagou A. Kontoghiorghe C. N. Mourouzidis L. Timoshnikov V. A. Polyakov N. E. (2020). Trying to solve the puzzle of the interaction of ascorbic acid and iron: Redox, chelation and therapeutic implications. Medicines 7 (8), 45. 10.3390/medicines7080045 32751493 PMC7460366

[B39] Koppenol W. H. Hider R. H. (2019). Iron and redox cycling. Do's and don'ts. Free Radic. Biol. Med. 133, 3–10. 10.1016/j.freeradbiomed.2018.09.022 30236787

[B40] Kwan P. Ho A. Baum L. (2022). Effects of deferasirox in alzheimer's disease and tauopathy animal models. Biomolecules 12 (3), 365. 10.3390/biom12030365 35327557 PMC8945800

[B41] Lane D. J. Bae D. H. Merlot A. M. Sahni S. Richardson D. R. (2015). Duodenal cytochrome b (DCYTB) in iron metabolism: An update on function and regulation. Nutrients 7 (4), 2274–2296. 10.3390/nu7042274 25835049 PMC4425144

[B42] Lane H. Y. Lin C. H. (2023). Diagnosing alzheimer's disease specifically and sensitively with pLG72 and cystine/glutamate antiporter SLC7A11 AS blood biomarkers. Int. J. Neuropsychopharmacol. 26 (1), 1–8. 10.1093/ijnp/pyac053 35986919 PMC9850657

[B43] Li B. Xia M. Zorec R. Parpura V. Verkhratsky A. (2021). Astrocytes in heavy metal neurotoxicity and neurodegeneration. Brain Res. 1752, 147234. 10.1016/j.brainres.2020.147234 33412145 PMC8999909

[B44] Lin Q. Shahid S. Hone-Blanchet A. Huang S. Wu J. Bisht A. (2023). Magnetic resonance evidence of increased iron content in subcortical brain regions in asymptomatic Alzheimer's disease. Hum. Brain Mapp. 44 (8), 3072–3083. 10.1002/hbm.26263 36929676 PMC10171513

[B45] Liu J. L. Fan Y. G. Yang Z. S. Wang Z. Y. Guo C. (2018). Iron and alzheimer's disease: From pathogenesis to therapeutic implications. Front. Neurosci. 12, 632. 10.3389/fnins.2018.00632 30250423 PMC6139360

[B46] Liu Y. Fang Y. Zhang Z. Luo Y. Zhang A. Lenahan C. (2022). Ferroptosis: An emerging therapeutic target in stroke. J. Neurochem. 160 (1), 64–73. 10.1111/jnc.15351 33733478

[B47] Logroscino G. Urso D. Savica R. (2022). Descriptive epidemiology of neurodegenerative diseases: What are the critical questions? Neuroepidemiology 56 (5), 309–318. 10.1159/000525639 35728570 PMC9677839

[B48] Long H. Z. Zhou Z. W. Cheng Y. Luo H. Y. Li F. J. Xu S. G. (2022). The role of microglia in alzheimer's disease from the perspective of immune inflammation and iron metabolism. Front. Aging Neurosci. 14, 888989. 10.3389/fnagi.2022.888989 35847685 PMC9284275

[B49] Luo Q. Q. Wang D. Yu M. Y. Zhu L. (2011). Effect of hypoxia on the expression of iron regulatory proteins 1 and the mechanisms involved. IUBMB life 63 (2), 120–128. 10.1002/iub.419 21360641

[B50] Ma H. Dong Y. Chu Y. Guo Y. Li L. (2022). The mechanisms of ferroptosis and its role in alzheimer's disease. Front. Mol. Biosci. 9, 965064. 10.3389/fmolb.2022.965064 36090039 PMC9459389

[B51] Malesza I. J. Bartkowiak-Wieczorek J. Winkler-Galicki J. Nowicka A. Dzięciołowska D. Błaszczyk M. (2022). The dark side of iron: The relationship between iron, inflammation and gut microbiota in selected diseases associated with iron deficiency anaemia-A narrative review. Nutrients 14 (17), 3478. 10.3390/nu14173478 36079734 PMC9458173

[B52] Mancardi D. Mezzanotte M. Arrigo E. Barinotti A. Roetto A. (2021). Iron overload, oxidative stress, and ferroptosis in the failing heart and liver. Antioxidants 10 (12), 1864. 10.3390/antiox10121864 34942967 PMC8698778

[B53] Mehkri Y. McDonald B. Sriram S. Reddy R. Kounelis-Wuillaume S. Roberts J. A. (2022). Recent treatment strategies in alzheimer's disease and chronic traumatic encephalopathy. Biomed. Res. Clin. Rev. 7 (3), 128. 10.31579/2692-9406/128 36743825

[B54] Menon V. Thomas R. Elgueta C. Horl M. Osborn T. Hallett P. J. (2019). Comprehensive cell surface antigen analysis identifies transferrin receptor protein-1 (CD71) as a negative selection marker for human neuronal cells. Stem cells 37 (10), 1293–1306. 10.1002/stem.3057 31381839 PMC6851846

[B55] Mesquita G. Silva T. Gomes A. C. Oliveira P. F. Alves M. G. Fernandes R. (2020). H-Ferritin is essential for macrophages' capacity to store or detoxify exogenously added iron. Sci. Rep. 10 (1), 3061. 10.1038/s41598-020-59898-0 32080266 PMC7033252

[B56] Mezzanotte M. Ammirata G. Boido M. Stanga S. Roetto A. (2022). Activation of the Hepcidin-Ferroportin1 pathway in the brain and astrocytic-neuronal crosstalk to counteract iron dyshomeostasis during aging. Sci. Rep. 12 (1), 11724. 10.1038/s41598-022-15812-4 35810203 PMC9271044

[B57] Misslinger M. Gsaller F. Hortschansky P. Müller C. Bracher F. Bromley M. J. (2017). The cytochrome b5 CybE is regulated by iron availability and is crucial for azole resistance in A. fumigatus. Metallomics 9 (11), 1655–1665. 10.1039/c7mt00110j 29072765

[B58] Monterey M. D. Wei H. Wu X. Wu J. Q. (2021). The many faces of astrocytes in alzheimer's disease. Front. neurology 12, 619626. 10.3389/fneur.2021.619626 PMC8438135 34531807

[B59] Mortberg M. A. Vallabh S. M. Minikel E. V. (2022). Disease stages and therapeutic hypotheses in two decades of neurodegenerative disease clinical trials. Sci. Rep. 12 (1), 17708. 10.1038/s41598-022-21820-1 36271285 PMC9587287

[B60] Moustafa A. A. Chakravarthy S. Phillips J. R. Gupta A. Keri S. Polner B. (2016). Motor symptoms in Parkinson's disease: A unified framework. Neurosci. Biobehav Rev. 68, 727–740. 10.1016/j.neubiorev.2016.07.010 27422450

[B61] Nemeth E. Ganz T. (2021). Hepcidin-ferroportin interaction controls systemic iron homeostasis. Int. J. Mol. Sci. 22, 6493. 10.3390/ijms22126493 34204327 PMC8235187

[B62] Nikseresht S. Bush A. I. Ayton S. (2019). Treating Alzheimer's disease by targeting iron. Br. J. Pharmacol. 176 (18), 3622–3635. 10.1111/bph.14567 30632143 PMC6715619

[B63] Nnah I. C. Wessling-Resnick M. (2018). Brain iron homeostasis: A focus on microglial iron. Pharm. (Basel) 11 (4), 129. 10.3390/ph11040129 PMC6316365 30477086

[B64] Nuñez M. T. Chana-Cuevas P. (2018). New perspectives in iron chelation therapy for the treatment of neurodegenerative diseases. Pharmaceuticals 11 (4), 109. 10.3390/ph11040109 30347635 PMC6316457

[B65] Onukwufor J. O. Dirksen R. T. Wojtovich A. P. (2022). Iron dysregulation in mitochondrial dysfunction and Alzheimer’s disease. Antioxidants 11, 692. 10.3390/antiox11040692 35453377 PMC9027385

[B66] Origa R. Cinus M. Pilia M. P. Gianesin B. Zappu A. Orecchia V. (2022). Safety and efficacy of the new combination iron chelation regimens in patients with transfusion-dependent thalassemia and severe iron overload. J. Clin. Med. 11 (7), 2010. 10.3390/jcm11072010 35407617 PMC8999930

[B67] Pandit R. Chen L. Götz J. (2020). The blood-brain barrier: Physiology and strategies for drug delivery. Adv. Drug Deliv. Rev. 165-166, 1–14. 10.1016/j.addr.2019.11.009 31790711

[B68] Parker J. B. Griffin M. F. Downer M. A. Akras D. Berry C. E. Cotterell A. C. (2023). Chelating the valley of death: Deferoxamine's path from bench to wound clinic. Front. Med. 10, 1015711. 10.3389/fmed.2023.1015711 PMC9975168 36873870

[B69] Peng Y. Chang X. Lang M. (2021). Iron homeostasis disorder and alzheimer's disease. Int. J. Mol. Sci. 22 (22), 12442. 10.3390/ijms222212442 34830326 PMC8622469

[B70] Peters D. G. Connor J. R. Meadowcroft M. D. (2015). The relationship between iron dyshomeostasis and amyloidogenesis in Alzheimer's disease: Two sides of the same coin. Neurobiol. Dis. 81, 49–65. 10.1016/j.nbd.2015.08.007 26303889 PMC4672943

[B71] Phelan J. J. Basdeo S. A. Tazoll S. C. McGivern S. Saborido J. R. Keane J. (2018). Modulating iron for metabolic support of TB host defense. Front. Immunol. 9, 2296. 10.3389/fimmu.2018.02296 30374347 PMC6196273

[B72] Piperno A. Pelucchi S. Mariani R. (2020). Inherited iron overload disorders. Transl. Gastroenterol. Hepatol. 5, 25. 10.21037/tgh.2019.11.15 32258529 PMC7063521

[B73] Poulos T. L. (2014). Heme enzyme structure and function. Chem. Rev. 114 (7), 3919–3962. 10.1021/cr400415k 24400737 PMC3981943

[B74] Raulin A. C. Doss S. V. Trottier Z. A. Ikezu T. C. Bu G. Liu C. C. (2022). ApoE in alzheimer's disease: Pathophysiology and therapeutic strategies. Mol. Neurodegener. 17 (1), 72. 10.1186/s13024-022-00574-4 36348357 PMC9644639

[B75] Ravalli F. Vela Parada X. Ujueta F. Pinotti R. Anstrom K. J. Lamas G. A. (2022). Chelation therapy in patients with cardiovascular disease: A systematic review. J. Am. Heart Assoc. 11 (6), e024648. 10.1161/JAHA.121.024648 35229619 PMC9075296

[B76] Rawat P. Sehar U. Bisht J. Selman A. Culberson J. Reddy P. H. (2022). Phosphorylated tau in Alzheimer’s disease and other tauopathies. Int. J. Mol. Sci. 23, 12841. 10.3390/ijms232112841 36361631 PMC9654278

[B77] Recalcati S. Cairo G. (2021). Macrophages and iron: A special relationship. Biomedicines 9 (11), 1585. 10.3390/biomedicines9111585 34829813 PMC8615895

[B78] Ryan F. Zarruk J. G. Lößlein L. David S. (2019). Ceruloplasmin plays a neuroprotective role in cerebral ischemia. Front. Neurosci. 12, 988. 10.3389/fnins.2018.00988 30670944 PMC6331473

[B79] Sadaf A. Hasan B. Das J. K. Colan S. Alvi N. (2018). Calcium channel blockers for preventing cardiomyopathy due to iron overload in people with transfusion-dependent beta thalassaemia. Cochrane Database Syst. Rev. 7 (7), CD011626. 10.1002/14651858.CD011626.pub2 29998494 PMC6513605

[B80] Samuni A. M. Afeworki M. Stein W. Yordanov A. T. DeGraff W. Krishna M. C. (2001). Multifunctional antioxidant activity of HBED iron chelator. Free Radic. Biol. Med. 30 (2), 170–177. 10.1016/s0891-5849(00)00459-7 11163534

[B81] Spence H. McNeil C. J. Waiter G. D. (2020). The impact of brain iron accumulation on cognition: A systematic review. PloS one 15 (10), e0240697. 10.1371/journal.pone.0240697 33057378 PMC7561208

[B82] Spotorno N. Acosta-Cabronero J. Stomrud E. Lampinen B. Strandberg O. T. van Westen D. (2020). Relationship between cortical iron and tau aggregation in Alzheimer's disease. Brain 143 (5), 1341–1349. 10.1093/brain/awaa089 32330946 PMC7241946

[B83] Sukhbaatar N. Weichhart T. (2018). Iron regulation: Macrophages in control. Pharm. (Basel) 11 (4), 137. 10.3390/ph11040137 PMC6316009 30558109

[B84] Sun L. Lin X. Pornprasert S. Lü X. Ran B. Lin Y. (2020). L-type calcium channel blockers decrease the iron overload-mediated oxidative stress in renal epithelial cells by reducing iron accumulation. Eur. J. Pharmacol. 886, 173513. 10.1016/j.ejphar.2020.173513 32898550

[B85] Tran D. DiGiacomo P. Born D. E. Georgiadis M. Zeineh M. (2022). Iron and alzheimer's disease: From pathology to imaging. Front. Hum. Neurosci. 16, 838692. 10.3389/fnhum.2022.838692 35911597 PMC9327617

[B86] Vaz M. Silvestre S. (2020). Alzheimer's disease: Recent treatment strategies. Eur. J. Pharmacol. 887, 173554. 10.1016/j.ejphar.2020.173554 32941929

[B87] Vela D. (2018). Low hepcidin in liver fibrosis and cirrhosis; a tale of progressive disorder and a case for a new biochemical marker. Mol. Med. 24 (1), 5. 10.1186/s10020-018-0008-7 30134796 PMC6016890

[B88] Wan W. Cao L. Kalionis B. Murthi P. Xia S. Guan Y. (2019). Iron deposition leads to hyperphosphorylation of tau and disruption of insulin signaling. Front. Neurol. 10, 607. 10.3389/fneur.2019.00607 31275224 PMC6593079

[B89] Wang F. Wang J. Shen Y. Li H. Rausch W-D. Huang X. (2022). Iron dyshomeostasis and ferroptosis: A new Alzheimer’s disease hypothesis? Front. Aging Neurosci. 14, 830569. 10.3389/fnagi.2022.830569 35391749 PMC8981915

[B90] Wang P. Wang Z. Y. (2017). Metal ions influx is a double edged sword for the pathogenesis of Alzheimer's disease. Ageing Res. Rev. 35, 265–290. 10.1016/j.arr.2016.10.003 27829171

[B91] Ward R. J. Dexter D. T. Crichton R. R. (2022). Iron, neuroinflammation and neurodegeneration. Int. J. Mol. Sci. 23 (13), 7267. 10.3390/ijms23137267 35806270 PMC9266893

[B92] Wilson D. M. Cookson M. R. Van Den Bosch L. Zetterberg H. Holtzman D. M. Dewachter I. (2023). Hallmarks of neurodegenerative diseases. Cell 186 (4), 693–714. 10.1016/j.cell.2022.12.032 36803602

[B93] Xie J. Van Hoecke L. Vandenbroucke R. E. (2022). The impact of systemic inflammation on alzheimer's disease pathology. Front. Immunol. 12, 796867. 10.3389/fimmu.2021.796867 35069578 PMC8770958

[B94] Xu H. Wang Y. Song N. Wang J. Jiang H. Xie J. (2018). New progress on the role of glia in iron metabolism and iron-induced degeneration of dopamine neurons in Parkinson's disease. Front. Mol. Neurosci. 10, 455. 10.3389/fnmol.2017.00455 29403352 PMC5780449

[B95] Yarali N. Fişgin T. Duru F. Kara A. Ecin N. Fitoz S. (2006). Subcutaneous bolus injection of deferoxamine is an alternative method to subcutaneous continuous infusion. J. Pediatr. Hematol. Oncol. 28 (1), 11–16. 16394886

[B96] Yiannikourides A. Latunde-Dada G. O. (2019). A short review of iron metabolism and pathophysiology of iron disorders. Med. (Basel) 6 (3), 85. 10.3390/medicines6030085 PMC6789448 31387234

[B97] Zacchi P. Belmonte B. Mangogna A. Morello G. Scola L. Martorana A. (2021). The ferroxidase hephaestin in lung cancer: Pathological significance and prognostic value. Front. Oncol. 11, 638856. 10.3389/fonc.2021.638856 34094919 PMC8170403

[B98] Zhang D. L. Ghosh M. C. Rouault T. A. (2014). The physiological functions of iron regulatory proteins in iron homeostasis - an update. Front. Pharmacol. 5, 124. 10.3389/fphar.2014.00124 24982634 PMC4056636

[B99] Zhang S. Xin W. Anderson G. J. Li R. Gao L. Chen S. (2022). Double-edge sword roles of iron in driving energy production versus instigating ferroptosis. Cell Death Dis. 13 (1), 40. 10.1038/s41419-021-04490-1 35013137 PMC8748693

[B100] Zhu Y. Chang J. Tan K. Huang S. K. Liu X. Wang X. (2021). Clioquinol attenuates pulmonary fibrosis through inactivation of fibroblasts via iron chelation. Am. J. Respir. Cell Mol. Biol. 65 (2), 189–200. 10.1165/rcmb.2020-0279OC 33861690

